# Solvents for Cholesterine, Etc.

**Published:** 1867-08

**Authors:** T. H. Buckler

**Affiliations:** Baltimore


### *Solvents for Cholesterine, etc.* By T. H. Buckler, M. D., of Baltimore.

It is believed that chloroform (terchloride of formyl) and succinate of the peroxide of iron will be found superior to any other agents as solvents for cholesteric fat, whether in or out of the living body. Chloroform taken into the stomach for the solution of gall-stones contained in the gall-bladder, and the continued use of succinate of iron to control the fatty or cholesteric diathesis and thereby prevent the formation of other calculi, have been invariably found by the writer trustworthy and successful after an experience of twenty years.

Turpentine and ether combined, as recommended by Durande, of Dijon, are the agents generally used as solvents of gall-stones. In regard to these remedies, we have only to say that a mass of cholesterine immersed in turpentine for three weeks had undergone no sensible loss of weight at the end of that time, and that however soluble this substance may be in ether, chloroform must always be found preferable, since it not only acts as the most rapid solvent, but at the same time produces the anæsthesia so necessary for the relief of the anguish invariably attendant on an acute attack of biliary colic.

In 1848 a mass of cholesterine of the size and shape of an ordinary sized hen's egg was taken from the gall-bladder of a woman who had died at the Baltimore Almshouse of some other affection. It was readily separated by gentle traction with the fingers into seventy-five irregularly quadrangular bodies about the size of an ordinary garden pea when dried. These were subjected, in separate vials, to every agent deemed capable of exerting on them a solvent influence. The masses immersed in the various acids, nitro-muriatic amongst others, had undergone no sensible loss of weight at the end of several weeks. Finally a mass, weighing several grains, immersed in Edinburgh chloroform, underwent solution in a few minutes, leaving only a friable refuse resembling the cinder of burned paper.

Not long afterwards we were consulted in the case of a married lady, aged 38, the mother of five children. She was stout and strong, and had always enjoyed good health up to the time of the attack, which was ushered in by paroxysms of pain in the right hypochondriac region, amounting at times to positive anguish. On examination, an irregular indurated tumor was felt through the walls of the abdomen, directly over the inferior margin of the liver. For some days this case was believed to be the cancer of the liver, but as the paroxysms of pain were followed by jaundice, and the facies of the patient was not that of cancer, a conjecture was formed that the indurated tumor in question might be produced by a distended gall-bladder filled to a greater or less extent with biliary calculi and protruding beyond the inferior margin of the liver. A teaspoonful of chloroform was given by the stomach every hour while the pain lasted, and a teaspoonful only after each meal for a period of five days longer, when the indurated tumor, before described, entirely subsided, affording unmistakable evidence that the gall-stones, none of which were found in the stools, had entirely dissolved out of the gall-bladder by the use of chloroform. About three months afterwards, this lady, who had enjoyed good health in the interval, was again seized with a paroxysm of anguish in the right hypochondriac region, owing evidently to a newly-formed gall-stone lodged in the trumpet-shaped mouth of the cystic duct. This being again dissolved by the use of chloroform, it was deemed desirable to administer some agent capable of controlling the cholesteric diathesis, and thereby prevent the further formation of biliary calculi. Seeing that the difficulty of dissolving gall-stones by any other agent than chloroform or ether grows out of the fact that cholesterine contains a very small amount of oxygen (from one and a half to two per cent., which is less than that of almost any other known substance), it seemed reasonable to suppose that a highly oxygenized compound would be found the best for attaining the object in view. Accordingly, succinic acid and peroxide of iron were selected, on account of oxygen contained in both of them. The hydrated succinate of the peroxide of iron was prepared by the eminent chemist Dr. David Stewart, of Baltimore. The lady, whose case we have just stated, made use of this salt as a permanent treatment to control the cholesteric diathesis, and thereby prevent the formation of gall-stones by destroying the raw material of which they are composed. She continued to take it for a period of six months, and resumed it afterwards in the following dose: ℞.—Hydrated succinate of the peroxide of iron, ℥ iss; water, f ℥ viss.—M. S.—A teaspoonful after each meal. She has had no recurrence of the attacks since she commenced the use of this salt, and enjoys excellent health at the present time. Three other cases treated with chloroform were promptly relieved.

It is above all other agents the remedy for this disease, performing as it does the double work of a solvent and an anæsthetic. In all of these cases the succinate of iron was used as permanent treatment after the chloroform had done its work. One other case terminated fatally, in which, in consultation, this treatment was advised but not carried out. The patient, a young and lovely married woman, Mrs. Z. P., residing at Sassafras, Eastern Shore of Maryland, aged twenty-one, was taken to a neighboring city to consult two deservedly eminent medical gentlemen, who told her she was much too young and full of health to have biliary calculi; and one of them gave her *Budd on Diseases of the Liver* to read as proof of the correctness of their opinion. They told her husband the previous attack was hysterical, and advised that she should live generously, and go to the opera every night by way of amusement. She did go to the opera each night, and eat a supper on returning to her hotel. The night of the last supper she was seized, shortly after going to bed, with pain in the right side, and continued in great anguish until ten o'clock the following morning, when she died. A post-mortem examination disclosed a gall-stone about the size and shape of a common Alpine strawberry, which her husband sent me.

After what has been demonstrated by H. Bence Jones and others in reference to the permeability of tissues, there is every reason to believe that a sufficient quantity of chloroform would pass into the gall-bladder through the intervening parts alone to dissolve out gall-stones, provided its use were continued a sufficient length of time; but when we reflect that chloroform is taken directly from the stomach into the current of the circulation, and that a large portion of it is carried directly to the acini of the liver, where, mingling with the newly-formed bile, it passes with it into the gall-bladder, it is easily seen that gall-stones may be dissolved with as much certainty as if they had been placed in a bath of cloroform outside of the living body.

The use of succinate of iron may also be extended with advantage to the treatment of leuco-phlegmatic subjects in whom there is a tendency to a redundance of fatty tissue, and when there is reason to suspect that a deposit of the great disorganizer, cholesteric fat, may be forming about the heart and arteries, or in other structures. The popular idea is that a man must be very well because he is very fat, when directly the reverse is often the case, adipose deposit being so frequently a form of structural degeneration of the tissues. It may be useful to persons having a tendency to obesity, and save them the necessity of carrying out the too rigid and often injurious Banting system of diet.

The writer is not aware that this salt has ever been prepared by any one but Dr. Stewart and his brother J. V. D. Stewart, of Baltimore, and it is perhaps important to state that it should be prepared in the hydrous state and kept constantly in a water-bath, for if dried it will never afterwards undergo a proper solution in water, but always form a gritty mixture.

This paper is only intended as a small contribution to the present knowledge of the subject, for the benefit of many better informed than the writer on this and all kindred topics, with the hope that they will communicate the results of their trials to the editor of the *American Journal of the Medical Sciences.*

